# Identification and functional characterization of *cis*-regulatory elements in the apicomplexan parasite *Toxoplasma gondii*

**DOI:** 10.1186/gb-2009-10-4-r34

**Published:** 2009-04-07

**Authors:** Nandita Mullapudi, Sandeep J Joseph, Jessica C Kissinger

**Affiliations:** 1 Department of Genetics, University of Georgia, East Green Street, Athens, Georgia, 30602, USA; 2 Center for Tropical and Emerging Global Diseases, University of Georgia, DW Brooks Drive, Athens, Georgia, 30602, USA; 3 Current address: Department of Pulmonary Medicine, Albert Einstein College of Medicine, Morris Park Ave, Bronx, New York, NY 10461, USA

## Abstract

**Background:**

*Toxoplasma gondii *is a member of the phylum Apicomplexa, which consists entirely of parasitic organisms that cause several diseases of veterinary and human importance. Fundamental mechanisms of gene regulation in this group of protistan parasites remain largely uncharacterized. Owing to their medical and veterinary importance, genome sequences are available for several apicomplexan parasites. Their genome sequences reveal an apparent paucity of known transcription factors and the absence of canonical *cis*-regulatory elements. We have approached the question of gene regulation from a sequence perspective by mining the genomic sequence data to identify putative *cis*-regulatory elements using a *de novo *approach.

**Results:**

We have identified putative *cis*-regulatory elements present upstream of functionally related groups of genes and subsequently characterized the function of some of these conserved elements using reporter assays in the parasite. We show a sequence-specific role in gene-expression for seven out of eight identified elements.

**Conclusions:**

This work demonstrates the power of pure sequence analysis in the absence of expression data or *a priori *knowledge of regulatory elements in eukaryotic organisms with compact genomes.

## Abstract 2

Mining of genomic sequence data of the apicomplexan parasite Toxoplasma gondii identifies putative cis-regulatory elements using a de novo approach.

## Background

*Toxoplasma gondii *is an obligate intracellular parasite belonging to the phylum Apicomplexa. The *T. gondii *genome is approximately 63 Mb, contains approximately 7,800 protein-encoding genes and has a GC content of 52%. Despite its reduced genome, the parasite exhibits a complex developmental life cycle wherein it is capable of switching between a rapidly dividing tachyzoite form and a quiescent bradyzoite form within the asexual stage of its life cycle [1]. During its asexual stage, it exhibits a wide host range, capable of infecting a variety of warm-blooded animals. Infection is of greater concern in AIDS or immunosuppressed patients, where it can lead to neurological, mental and ocular defects. It is also responsible for human birth defects and spontaneous abortion as a result of trans-placental transmission in infected pregnant women [2,3]. Given its wide host-range and medical importance, understanding fundamental processes of gene regulation is important for developing methods aimed at controlling infection and disease.

There are many levels at which organisms can control gene expression, including chromatin-mediated modifications, transcriptional and post-transcriptional regulation, and post-translational regulation [4,5]. Transcription factors that mediate transcriptional regulation can be sequence-specific DNA-binding proteins that are involved in gene-specific regulation, or more general RNA polymerase II components that are required for transcription initiation. Promoter organization in unicellular eukaryotes such as *Saccharomyces cerevisiae *is composed of a bi-partite structure consisting of a core promoter located close to the start of transcription and upstream activator sequences that contain binding sites for sequence-specific transcription factors present a few hundred base pairs away. In metazoans, additional, more distal elements, such as enhancers and insulator elements, provide for more specific fine-tuning of gene-regulation [6]. Very little is known about how *T. gondii *and other apicomplexan parasites regulate their genes. A relatively small number of gene-specific studies in *T. gondii *have identified non-canonical *cis*-regulatory elements indicative of a bi-partite promoter organization that were found to play a role in downstream gene expression [7,8]. Preliminary surveys of the complete genome sequence have revealed a paucity of known specialized transcriptional factors encoded in the genome [9]. Recent studies have focused on dissecting the developmental signals responsible for inter-conversion between the tachyzoite and bradyzoite developmental stages and the preferential gene expression that characterizes these stages. To this end, the study of stage-specific genes and their promoters [10-12] has revealed the presence of *cis*-regulatory elements in the promoter region that are responsible for preferential gene expression in different life cycle stages. Large-scale analyses of gene expression from key developmental life cycle stages [13] point to the absence of chromosomal clustering of co-expressed genes, and the presence of unique stage-specific mRNAs in each developmental stage. However, promoter organization and the presence of specialized transcription factors for their recognition remain largely unexplored areas. The medical importance combined with the evolutionary divergence of the apicomplexan parasites relative to model organisms has motivated a rapidly growing collection of genome sequencing efforts for this group.

Sequence information provides us with a starting point to identify *cis*-acting signals in the genome and to uncover underlying gene-regulatory mechanisms. Sequence analysis to identify conserved *cis*-regulatory signals is typically augmented by at least one of two types of information: the organization of regulons and known sequences of conserved transcription factor binding sites, or large-scale gene expression information (for example, from microarray studies), that provide data sets of co-regulated genes within which conserved transcription factor binding sites can be identified [14]. Known canonical eukaryotic *cis*-elements have not yet been reported in *T. gondii*. In the absence of this starting information, we have adopted a *de novo *approach to identify conserved sequence elements that could serve as putative *cis*-regulatory elements. We have then experimentally verified the role for these candidate elements in the parasite, establishing their role in gene expression. Our study includes four different groups of genes that share parasite-specific or metabolic functions. We describe a computational framework for the identification of novel *cis*-regulatory elements in eukaryotic non-model systems, particularly those with reduced genomes and relatively small intergenic regions.

## Results and discussion

We analyzed four different functional groups of genes for the presence of conserved, over-represented upstream sequence motifs within each group. The choice of seed genes was based on the hypothesis that genes that share a common function or operate in the same biochemical pathway should be co-regulated and possess common upstream regulatory elements. We used MEME (Multiple Em for Motif Elicitation) [15], a *de novo *pattern-finding algorithm to detect such motifs within each group of genes. We tested the functional significance of top candidate motifs by mutagenizing them in their native promoter context and measuring subsequent reporter gene expression (see Materials and methods). We find that different groups of genes share different over-represented motifs and no global motif emerges from our studies to be shared by all groups. The results of pattern finding and accompanying experimental evidence establish the biological role of the motifs considered in this study.

### Genes involved in glycolysis

*T. gondii*, like *Eimeria tenella *and *Cryptosporidium parvum*, uses glucose as its main source of energy in its rapidly dividing tachyzoite stage [16]. Phylogenetic analyses have shown that two of the glycolytic genes in *T. gondii*, enolase and glucose-6-phosphate isomerase, are closely related to their corresponding homologs in plants, suggesting that they were acquired and potentially suitable as drug targets due to their distinct evolutionary origin [17]. Glycolysis has also been actively studied with respect to stage differentiation in *T. gondii*. Three key glycolytic enzymes - glucose-6-phosphate isomerase [ToxoDB:76.m00001], lactate dehydrogenase (LDH) and enolase (ENO) [ToxoDB:59.m03410] - exhibit developmentally regulated expression [18]. Stage-specific cDNAs have been isolated that encode distinct isoforms of LDH: *LDH1 *(tachyzoite) and *LDH2 *(bradyzoite) [19]. Experimental evidence based on the detection of their respective mRNA and protein products indicates that *LDH1 *is post-translationally repressed while *LDH2 *is transcriptionally induced in bradyzoites [19]. Similarly, stage-specific cDNAs have also been isolated for distinct forms of ENO: *ENO1 *(bradyzoite) and *ENO2 *(tachyzoite) [20]. Stage-specific expression of the two enolases is brought about by the presence of specific *cis*-regulatory elements in the promoter regions of these genes [10]. The regulation of the genes involved in glycolysis presents an intriguing case study from developmental, evolutionary and regulatory perspectives.

**Figure 1:**
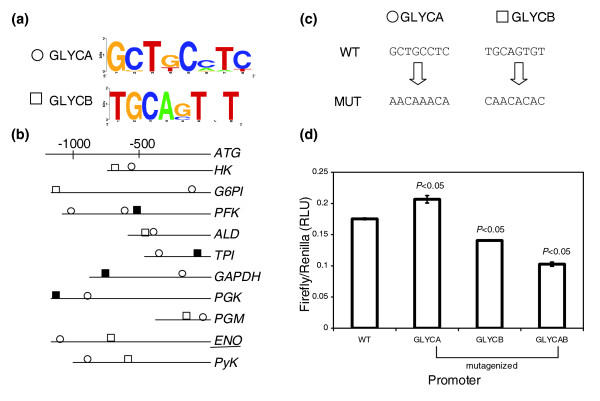
Candidate motifs identified upstream of glycolytic genes, upstream location, site-directed mutagenesis and results of reporter assays. Motifs GLYCA and GLYCB act in concert to influence gene-expression from the *Eno2 *promoter. **(a) **Sequence logos represent the consensus sequence for each candidate motif. The y-axis represents information content at each position. **(b) **Occurrences and positions of the motifs in the promoter region relative to the translational start site of each gene. The gene names are abbreviated as shown in Table 1. The underlined gene name indicates the representative promoter used in reporter assays. Motif GLYCA, found in both *E. tenella *and *T. gondii*, is denoted by a circle and motif GLYCB, exclusive to *T. gondii*, is denoted by a square. Solid shapes denote motifs on the opposite strand. **(c) **The wild-type (WT) motifs and their mutagenized (MUT) versions in the representative promoter are represented. **(d) **The graphs depict luciferase activity as ratios of firefly:renilla activity in relative luciferase units (RLU) from the different constructs containing either WT or mutagenized versions of GLYCA, GLYCB, or both motifs. All luciferase readings are relative to an internal control (α-tubulin-renilla). Error bars represent standard error calculated across the means of three independent electroporations. *p*-values describe the probability that the difference in expression between the WT and mutagenized promoters may be due to chance.

We analyzed the upstream sequences of 11 genes involved in tachyzoite glycolysis to identify conserved, over-represented sequence motifs (Table 1). We report the analysis of two candidate motifs here: motif GLYCA, also found upstream of six orthologs in *E. tenella*, and motif GLYCB, found exclusively in *T. gondii*. These motifs were not reported in the aforementioned studies on stage-specific regulation of the enolase gene [18]. Motif GLYCA, represented by the consensus 5'GCTKCMTY (Figure 1a) is an 8 bp well-conserved sequence occurring at least once per sequence on the forward strand (Figure 1b). It does not show significant positional conservation, but motifs found upstream of orthologs in *E. tenella *are found to be 100% conserved in sequence to their counterpart in *T. gondii*. Motif GLYCA is not found in the upstream regions of the bradyzoite isoforms of the stage-specific glycolytic genes (*ENO2 *and *LDH1*). Motif GLYCB is also an 8 bp motif represented by the consensus sequence 5'TGCASTNT (Figure 1a), with 6 of 8 bases conserved in more than 90% of the occurrences. This motif is present once per sequence and can occur on either strand (Figure 1b). Motif GLYCB was also found in the upstream regions of the bradyzoite-specific copies of enolase and LDH (data not shown).

**Table 1:** List of genes used in this study

Symbol	Gene name	Ortholog	ToxoDB ID	Promoter length (bp)
**Gylcolysis**				
*HK*	*Hexokinase*	+	57.m00001	900
*G6PI*	*Glucose-6-phosphate-isomerase*	+	76.m00001	2,000
*PFK*	*Phosphofructokinase*		49.m03242	2,000
*ALD*	*Aldolase*	+	46.m00002	1,370
*TPI*	*Triose-phosphate-isomerase*	+	42.m00050	2,000
*GAPDH*	*Glyceraldehydye-3-phosphate dehydrogenase*	+	80.m00003	2,000
*PGK*	*Phosphoglycerate kinase*		641.m000193	2,000
*PGM*	*Phosphoglucomutase*		113.m00016	1,500
*ENO*	Enolase*	+	59.m03410	2,000
*PyK*	*Pyruvate kinase*		55.m00007	1,500
				
**Nucleotide metabolism**				
*AK*	*Adenosine kinase*	+	50.m00018	2,000
*CTPS*	*Cytidine synthase*	+	129.m00261	2,000
*DCDA*	*Deoxycytidine deaminase*	+	8.m00191	2,000
*DHFR-TS*	*Dihydrofolate reducatase-thymidine synthase*		50.m00016	2,000
*GMPS*	*Guanidine monophosphate synthase*	+	44.m00023	2,000
*RDPR*	*Ribonucelotide diphosphate reductase*		83.m00003	2,000
*UPRT*	Uracil phosphoribosyl transferase*		583.m00018	2,000
*AT*	*Adenosine transporter*		49.m00004	2,000
				
**Micronemal proteins**				
*MIC1*	*Microneme 1*	+	80.m00012	1,500
*MIC2*	*Microneme 2*	+	20.m00002	2,000
*MIC3*	*Microneme 3*		641.m00002	2,000
*MIC4*	*Microneme 4*	+	25.m00006	2,000
*MIC5*	*Microneme 5*	+	65.m00002	2,000
*MIC6*	*Microneme 6*	+	38.m00003	2,000
*MIC7*	*Microneme 7*		55.m00014	2,000
*MIC8*	Microneme 8*		50.m00002	2,000
*MIC9*	*Microneme 9*		49.m03396	2,000
*MIC10*	*Microneme 10*	+	50.m00010	2,000
*MIC11*	*Microneme 11*		20.m05914	2,000
*M2AP*	*Microneme-2-associated protein*		33.m00006	2,000
				
**Ribosomal proteins**				
*RPS29*	*Ribosomal protein S29*		49.m03285	800
*RPS38*	*Ribosomal protein S38*		44.m04616	1,000
*RPS3*	*Ribosomal protein S3*		44.m04669	1,000
*RPS13*	*Ribosomal protein S13*		59.m03516	1,000
*RPL9*	Ribosomal protein L9*		76.m00009	1,200
*RPS25*	*Ribosomal protein S25*		44.m00003	1,300
*RPS10*	*Ribosomal protein S10*		64.m00338	700
*RPL25*	*Ribosomal protein L25*		55.m00189	1,000

The list of genes and the lengths of their upstream regions that were used in the studies to identify regulatory motifs. A plus sign in the Ortholog column indicates that a corresponding ortholog in *E. tenella *was obtained and added to the search. Representative genes used in mutagenesis and expression analyses are denoted by an asterisk.

Mutagenesis of GLYCA to the sequence 5'AACAAACA in the *ENO2 *promoter resulted in a small increase in promoter activity. Mutagenesis of GLYCB to the sequence 5'CAACACAC within the ENO2 promoter resulted in a small decrease in promoter activity (Figure 1c, d). However, when both motifs were mutagenized, a larger decrease in promoter activity was seen. These results are complex in comparison to patterns seen with motifs for other groups of genes (see below). It must be noted that the changes in expression levels caused by mutagenizing each individual sequence in the ENO2 promoter are of small magnitude, but statistically significant. It is possible that the effects of mutagenizing each motif are not very severe in their effect, while the double mutant shows a large decrease in reporter expression, indicating a definite role for both of these motifs, in concert, to affect downstream gene expression. An alternative scenario to explain this result is one in which mutagenesis of GLYCA gives rise to a chimeric motif that enhances downstream gene-expression only in the presence of wild-type (WT) GLYCB. The strong evolutionary conservation of motif GLYCA in *E. tenella *and the significant decrease in reporter activity in the double mutant lend support to their role in regulating gene expression. Further experiments are needed to fully resolve these intriguing results.

### Genes involved in nucleotide biosynthesis and salvage

**Figure 2:**
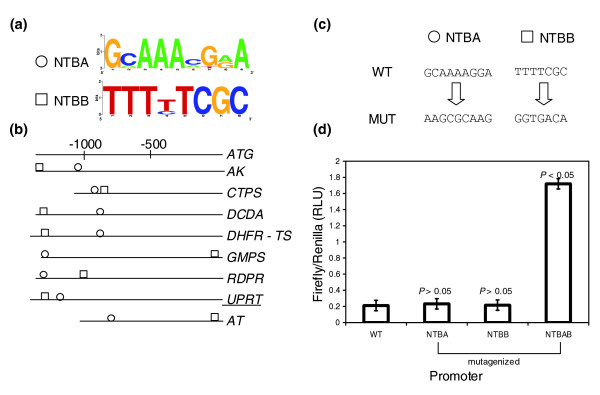
Candidate motifs identified upstream of the nucleotide biosynthetic genes, upstream location, site-directed mutagenesis and results of reporter assays. Motifs NTBA and NTBB show redundancy in function by negatively affecting gene expression from the UPRT promoter among the nucleotide metabolism genes. **(a) **Sequence logos represent the consensus sequence for each candidate motif. The y-axis represents information content at each position. **(b) **Occurrences and positions of the motifs in the promoter region relative to the translational start site of each gene. The gene names are abbreviated as shown in Table 1. The underlined gene name indicates the representative promoter used in reporter assays. Motif NTBA, found in both *E. tenella *and *T. gondii*, is denoted by a circle and motif NTBB, exclusive to *T. gondii*, is denoted by a square. **(c) **The WT motifs and their mutagenized (MUT) versions in the representative promoter are represented. **(d) **The graphs depict luciferase activity as ratios of firefly:renilla activity in relative luciferase units (RLU) from the different constructs containing either WT or mutagenized versions of NTBA, NTBB, or both motifs. All luciferase readings are relative to an internal control (α-tubulin-renilla). Error bars represent standard error calculated across the means of three independent electroporations. *p*-values describe the probability that the difference in expression between the WT and mutagenized promoters may be due to chance.

Purines and pyrimidines are the building blocks of nucleic acids in living cells. All protozoan parasites examined thus far are unable to synthesize purines *de novo *and depend upon salvage enzymes to obtain purines from the host [21]. Most protists, however, possess a full set of *de novo *pyrimidine biosynthesis enzymes, with one exception, *C. parvum*, which has lost the *de novo *pathway and evolved to also salvage pyrimidines from the host cell [22]. Enzymes involved in nucleotide metabolism in protozoan parasites can serve as promising drug targets because they are essential to the parasite's survival and are also evolutionarily distinct from host enzymes in some cases [22]. In *T. gondii*, it was found that *de novo *pyrimidine biosynthesis is essential for the virulence of the parasite [23]. We examined eight genes encoding enzymes involved in nucleotide biosynthesis and salvage in *T. gondii *and selected two conserved motifs found in their upstream regions as candidates for experimental validation. Motif NTBA is an A-rich 9 bp motif represented by the consensus 5'GCAAAMGRA (Figure 2a). It is very well conserved in four orthologs in *E. tenella*. Motif NTBA is present only once upstream of each gene and is always found on the positive strand. It is primarily located at 1,000-1,500 bp upstream of the translation start (Figure 2b). Motif NTBB is an 8 bp long T-rich motif and is exclusive to *T. gondii*. It is represented by the consensus sequence 5'TTTYTCGC (Figure 2a) and is also found only once upstream of each gene on the forward strand. The two motifs are typically present within 300-400 bp of each other (Figure 2b).

To establish the biological significance of these motifs, we mutagenized NTBA to the sequence 5'AAGCGCAAG and NTBB to the sequence 5'GTGTGTG (Figure 2c). Mutagenesis of either of these motifs individually in the promoter of the gene encoding uracil phosphoribosyl transferase (UPRT) [ToxoDB:583.m00018] showed no significant change in promoter activity. Mutagenesis of both motifs within the UPRT promoter resulted in a seven-fold increase in reporter gene-expression, indicating that the two motifs function in repressing gene-expression and possibly possess redundancy in function (Figure 2d).

### Genes encoding micronemal proteins

**Figure 3:**
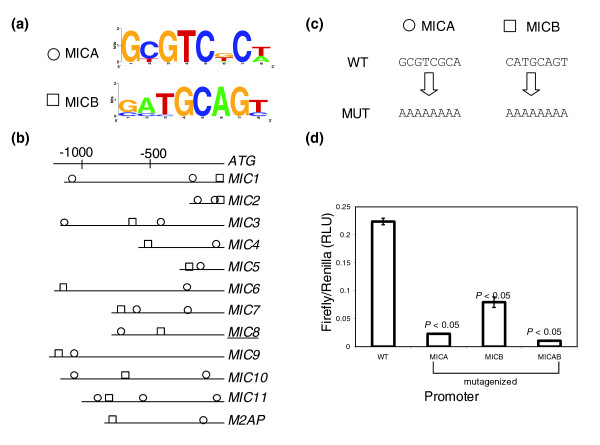
Candidate motifs identified upstream of the micronemal protein-encoding genes, upstream location, site-directed mutagenesis and results of reporter assays. Motifs MICA and MICB display an additive effect in the regulation of the gene encoding microneme 8. **(a) **Sequence logos represent the consensus sequence for each candidate motif. The y-axis represents information content at each position. **(b) **Occurrences and positions of the motifs in the promoter region relative to the translational start site of each gene. The gene names are abbreviated as shown in Table 1. The underlined gene name indicates the representative promoter used in reporter assays. Motif MICA, found in both *E. tenella *and *T. gondii*, is denoted by a circle and motif MICB, exclusive to *T. gondii*, is denoted by a square. **(c) **The WT motifs and their mutagenized (MUT) versions in the representative promoter are represented. **(d) **The graphs depict luciferase activity as ratios of firefly:renilla activity in relative luciferase units (RLU) from the different constructs containing either WT or mutagenized versions of MICA, MICB, or both motifs. All luciferase readings are relative to an internal control (α-tubulin-renilla). Error bars represent standard error calculated across the means of three independent electroporations. *p*-values describe the probability that the difference in expression between the WT and mutagenized promoters may be due to chance.

Micronemes are secretory organelles found in apicomplexan parasites and serve as compartments for the storage and trafficking of micronemal proteins, a family of proteins that function as ligand for host-cell receptors [24]. These proteins play a very important role in the active process of host-cell adhesion and invasion during the parasite life cycle. We analyzed the upstream sequences of 12 microneme protein-encoding genes in *T. gondii *and corresponding upstream sequences of four orthologs in *E. tenella*. We identified two well-conserved sequence motifs in this data set that we subsequently selected for further experimental characterization. Motif MICA is an 8 bp motif represented by the consensus sequence 5'GCGTCDCW (Figure 3a). It is found at least twice in the majority of the upstream regions occurring on either strand and does not show conservation of position relative to the translational start site (Figure 3b). This motif was also found upstream of *E. tenella *micronemal protein genes. In the reverse orientation, this motif closely resembles the 5'WGAGACG motif that has been identified in previous studies to function as a regulatory element in several promoters of *T. gondii *[8]. Motif MICB is an 8 bp motif with the very well conserved sequence 5'SMTGCAGY (Figure 3a); the core 'TGCA' nucleotides are conserved in 100% of occurrences. This motif occurs once upstream in all 11 micronemal protein genes in *T. gondii*, but was not found in the corresponding orthologs in *E. tenella*. It does not show conservation of position relative to the translational start site, and is always found on the forward strand (Figure 3b).

To characterize the functional significance of these conserved motifs, each was mutagenized to an 8 bp polyA sequence (5'AAAAAAAA; Figure 3c). The mutagenesis of motif MICA in the Mic8 (Micronemal protein 8) [ToxoDB: 50.m00002] promoter led to a tenfold reduction in reporter activity, and the mutagenesis of motif MICB led to a threefold reduction in reporter expression. When both MICA and MICB were mutagenized in the same promoter, it had a dramatic effect on promoter activity (the raw value of firefly expression levels (440 units) was comparable to that of non-transfected cells (386 units) (Figure 3d)). From these data, we infer that both MICA and MICB act positively to enhance gene expression from the *Mic8 *promoter, and together exert an additive effect on downstream gene-expression, as is indicated by the loss of expression when both MICA and MICB are mutagenized (Figure 3d).

### Ribosomal protein encoding genes

**Figure 4:**
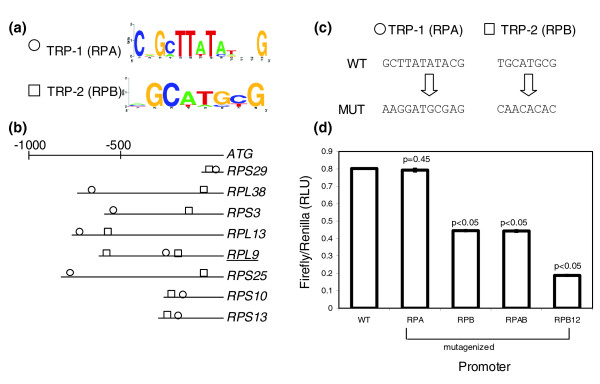
Candidate motifs identified upstream of the ribosomal protein genes, upstream location, site-directed mutagenesis and results of reporter assays. Motif RPA (TRP-1) does not influence reporter activity, and motif RPB (TRP-2) acts as an enhancer of gene-expression from the RPL9 promoter. **(a) **Sequence logos represent the consensus sequence for each candidate motif. The y-axis represents information content at each position. **(b) **Occurrences and positions of the motifs in the promoter region relative to the translational start site of each gene. The gene names are abbreviated as shown in Table 1. The underlined gene name indicates the representative promoter used in the reporter assays. **(c) **The WT motifs and their mutagenized (MUT) versions in the representative promoter are represented. **(d) **The graphs depict luciferase activity as ratios of firefly:renilla activity in relative luciferase units (RLU) from the different constructs containing either WT or mutagenized versions of RPA, RPB, both motifs or both copies of motif RPB. All luciferase readings are relative to an internal control (α-tubulin-renilla). Error bars represent standard error calculated across the means of three independent electroporations. *p*-values describe the probability that the difference in expression between the WT and mutagenized promoters may be due to chance.

Examination of stage-specific expressed sequence tag libraries in *E. tenella *and *T. gondii *indicates that the coccidia regulate *de novo *ribosome biosynthesis at the transcriptional level [25]. In a recent study [26] the authors examined a large set of cytoplasmic ribosomal proteins in *T. gondii *(79 genes in all) and describe the presence of two well-conserved motifs, TRP-1 (motif RPA; 5'CGGCTTATATTCG) and TRP-2 (motif RPB; 5'YGCATGCR) (Figure 4a) identified by MEME in all promoters. The sequence of TRP-2 (RPB) is similar to the 8 bp element 5'TGCATGCA reported to be overrepresented in the non-coding regions of the apicomplexans *C. parvum*, *T. gondii *and *E. tenella *[27]. This sequence is also similar to one of the binding sites of the AP2-domain containing transcription factors as inferred from protein-based microarray studies conducted in *P. falciparum *[28]. In a study of the promoter strengths of eight of the ribosomal protein genes, no correlation could be found between multiple occurrences of one or both motifs and promoter strength in the eight promoters [29]. However, the biological function of these motifs was not reported. We conducted analyses on a subset of these genes (eight promoters) and also recovered the motifs TRP-1 (RPA) and TRP-2 (RPB) as described by van Poppel *et al*. [29] (Figure 4b). We mutagenized these motifs in our analyses to ascertain if they functioned in a sequence-specific manner to affect promoter activity.

Motif TRP-1 (RPA) in the *RPL9 *(Ribosomal protein L9) promoter [ToxoDB:76.m00009] was mutagenized to the sequence 5'CGAAGTATGCGAG (retaining the WT sequence at 3 of the 13 nucleotide positions due to mutagenesis challenges presented by the length of this motif) and motif TRP-2 (RPB), which occurs twice in the *RPL9 *promoter, was mutagenized at both sites (singly and jointly) to the sequence 5'TAAATAAA (Figure 4c). TRP-1 (RPA) did not affect reporter expression when mutagenized individually or in combination with TRP-2 (RPB). This observation may be attributed to the fact that not all of the bases in this motif were mutagenized, indicating that the three WT positions might be crucial and sufficient for the function of this motif or that this motif may serve a function during a different stage of development or not serve a function related to gene expression. These results warrant further examination. Mutagenesis of one of the copies of motif RPB resulted in a 50% reduction in promoter activity, while mutagenesis of both the copies of RPB caused a 75% reduction in gene expression relative to the WT promoter (Figure 4d). These data indicate that TRP-2 (RPB) enhances gene expression from the *RPL9 *promoter; the presence of additional copies of this motif likely confers additional strength to the promoter.

### Genome-wide occurrences of candidate motifs

**Figure 5:**
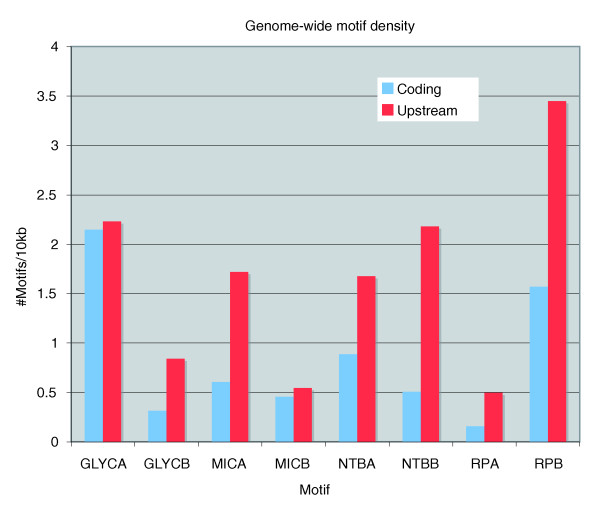
Genome-wide occurrences of candidate motifs. Most of the candidate motifs with verified biological function are over-represented within upstream regions. Motif density is plotted as number of motifs per 10 kb for each data set - upstream sequences (red) and coding sequences (blue) (Table 2) - on the y-axis for each candidate motif on the x-axis.

We examined the occurrences of each of the motifs to determine if there was over-representation within upstream regions relative to coding regions. Table 2 lists the genome-wide occurrences of each of the candidate motifs within the upstream and the coding regions of the genome, respectively, as computed by MAST (Motif Analysis and Search Tool) [15]. In order to normalize for the different sizes of the two data sets, the motif count is represented as number of motifs per 10 kbp (motif density). Of the eight candidate motifs selected in this study, the RPB (TRP-2) motif (5'YGCATGCR) has the highest occurrence within upstream regions, 4,030 occurrences upstream of 1,311 genes. When normalized to the total size of each database (upstream or coding), the candidate motifs (except GLYCA and MICB) were found to be significantly (two- to four-fold) over-represented (*p *< 0.001) in the upstream regions relative to the coding regions (Table 2, Figure 5).

**Table 2:** Genome-wide occurrences of each candidate motif within coding and upstream regions

	Upstream			Coding			
			
Motif	Number of genes	Number of motifs	Number of motifs/10 kb	Number of genes	Number of motifs	Number of motifs/10 kb	*p*-value
GLYCA	885	2608	2.23	956	3618	2.14	0.0538
GLYCB	418	982	0.84	201	531	0.31	< 0.001
MICA	734	2010	1.72	435	1019	0.6	< 0.001
MICB	223	637	0.54	290	769	0.46	0.0026
NTBA	658	1959	1.67	418	1495	0.89	< 0.001
NTBB	1100	2548	2.18	359	852	0.5	< 0.001
RPA	368	581	0.49	145	262	0.15	< 0.001
RPB	1311	4030	3.45	810	2648	1.57	< 0.001

The number of occurrences of each motif and the genes containing them in the whole genome Motif density (number of motifs per 10 kb) was computed using MAST to search position weight matrix profiles of each motif against custom built databases (upstream regions (11,685,162 bp) and coding regions (16,862,741 bp)).

We calculated the expected frequency of motifs within the upstream and coding regions based on the motif length, degeneracy and the composition and size of the database (Materials and methods). The expected occurrences of most of the motifs are almost equal in both databases (upstream and coding) because of the similarity in size and nucleotide composition of the two databases. The motifs are not found to occur at a significantly greater frequency than expected, exceptions being NTBA, which is found at a higher frequency than expected (*p *< 0.05) within the upstream and coding regions, and motifs NTBB and RPA, which are found at frequencies higher than expected in the coding regions only (Table 3 in Additional data file 1).

**Table 3:** Gene Ontology categories significantly enriched among motif-containing genes

Motif	GO category	Description	*p*-value	Adjusted *p*-value
GLYCB	GO:0016530	Metallochaperone activity	1.00E-05	0.0004
				
MICB	GO:0016530	Metallochaperone activity	1.00E-05	0.0004
				
NTBA	GO:0022414	Reproductive process	1.00E-06	0.0001
NTBA	GO:0016530	Metallochaperone activity	1.00E-05	0.0004
				
NTBB	GO:0002376	Immune system process	1.00E-06	0.0002
NTBB	GO:0009987	Cellular process	0.0005	0.0110
NTBB	GO:0009055	Electron carrier activity	0.0025	0.0371
NTBB	GO:0008152	Metabolic process	0.0026	0.0367
NTBB	GO:0030234	Enzyme regulator activity	0.0030	0.0400
				
RPA	GO:0045735	Nutrient reservoir activity	1.00E-06	0.0001
RPA	GO:0005840	Ribosome	1.45E-06	0.0001
RPA	GO:0005198	Structural molecule activity	3.65E-06	0.0002
RPA	GO:0006412	Translation	0.0001	0.0039
RPA	GO:0009987	Cellular process	0.0004	0.0096
RPA	GO-0043226	Organelle	0.0005	0.0110
RPA	GO-0032991	Macromolecular complex	0.0005	0.0107
RPA	GO:0051234	Establishment localization	0.0008	0.0148
RPA	GO:0051179	Localization	0.0008	0.0156
				
RPB	GO-0044421	Extracellular region part	1.00E-07	3.52E-05
RPB	GO:0022414	Reproductive process	1.00E-06	0.0001
RPB	GO:0016530	Metallochaperone activity	1.00E-05	0.0003
RPB	GO:0006412	Translation	0.0001	0.0020
RPB	GO:0005840	Ribosome	0.0003	0.0074
RPB	GO-0043226	Organelle	0.0010	0.0172
RPB	GO:0009987	Cellular process	0.0018	0.0285
RPB	GO:0005198	Structural molecule activity	0.0018	0.0282

Significantly enriched GO categories (adjusted *p*-value < 0.05) for motif-containing genes. The results are listed by each motif and ordered based on their Benjamini Hochberg adjusted *p*-value. A good correlation is observed between the functional pathways associated with the seed genes and the GO category that is over-represented in the corresponding motif-containing genome-wide gene sets in the case of motifs NTBB, RPA and RPB.

Thus, while most of the regulatory motifs are present at a slightly higher frequency in the upstream regions when compared to the coding regions, they do not occur at a higher frequency than expected in either upstream or coding regions. These analyses highlight the limitations of approaches that use statistical overrepresentation of motifs as a reliable and sufficient property to identify biologically relevant motifs. It is possible that a functional regulatory motif may not be detectable by sequence alone. The surrounding sequence context and other still elusive signals may be involved in enabling it to function as a regulatory motif.

To examine enrichment of specific Gene Ontology (GO) categories among all genes containing any of the eight candidate upstream motifs, we retrieved first-level GO annotations for all of the motif-containing genes (Table 2 in Additional data file 1) for each of the three main GO categories: 'cellular component', 'molecular function' and 'biological process'. We also included lower level GO annotation IDs for the specific pathways/functional groups included in this study (Materials and methods). Table 4 in Additional data file 1 lists the GO categories that were significantly enriched within the motif-containing gene sets. Some of the motif-containing gene sets are also enriched in GO terms related to the corresponding function/pathway used to initially identify the motif, indicating that the regulatory motif may indeed be a subset-specific or pathway-specific motif. On the other hand, some motif-containing gene sets do not show enrichment for a particular GO category, but rather to a more general, functional classification. For example, genes containing the motifs discovered in the analysis of ribosomal protein-coding genes (RPA and RPB) are enriched in annotated higher-level GO categories such as organelle and regulation of biological process. This indicates that a large number of genes that contain the RPA (TRP-1) and RPB (TRP-2) motifs can be assigned to ribosome or translational-specific functions, indicating a broad subset specificity for this motif. Genes that contain the MICA or MICB motifs do not show any GO category enrichment, indicating a more general role for these upstream motifs. When deeper-level GO annotations for particular processes (such as 'ribosome' [GO:0005840]) are enumerated among the motif-containing genes, we find that the genome-wide lists of genes that contain RPA and RPB motifs are also enriched in corresponding GO categories ('ribosome' and 'translation'), indicating an even stronger specific association of these motifs with the corresponding processes (Table 3).

### General discussion

Promoter organization in *T. gondii *has been studied in a few genes thus far [7,8,10,11]. In these studies, it has been observed that a gene-proximal region is necessary for minimal gene expression and additional upstream sequence helps to enhance expression from the same promoter. However, very little is known about the mechanism of gene regulation and the prevalence and type of transcriptional signals and regulatory apparatus in this organism. Analyses of genome sequences and individual gene-specific experiments point out two deviations from what has been observed in other model eukaryotes. First, canonical eukaryotic promoter elements such as the TATA box have not been found in *T. gondii *promoter regions [8], although a highly divergent TATA binding protein has been reported [9]. Furthermore, there is a stark paucity of known specialized transcription factors encoded in the genome [9]. A similar scenario is seen in two other apicomplexan parasites, *P. falciparum *and *C. parvum *[30,31]. This paradox can be explained in two ways: these organisms do not employ a specialized transcriptional apparatus to regulate their genes; or a specialized transcriptional machinery exists but is so divergent from known eukaryotic counterparts that its components cannot be detected by simple similarity-based searches. Recent studies have shown that the *T. gondii *genome encodes a rich repertoire of histone-modifying enzymes, and epigenetic regulation has been purported to be responsible for stage-switching in the parasite [32,33]. More recently, chromatin immunoprecipitation (ChIP)-on-chip experiments conducted on 1% of the *T. gondii *genome reveal a strong association between specific histone modification marks and active promoter regions [34]. It is likely that histone-mediated regulation is responsible for regulation of genes to a sizeable extent in *T. gondii*. Serial analysis of gene expression (SAGE) studies of genes expressed during key life-cycle stages [13] have shown that the mRNA pool of *T. gondii *is highly dynamic and gene expression is controlled in a time- and stage-dependent manner. These studies have also shown that co-expressed genes in *T. gondii *do not cluster in the genome with respect to chromosomal location. Searches of the *Plasmodium *genome sequence for transcription factors using secondary structure similarity have revealed the presence of putative transcription factors that were missed in simple sequence-based searches [35]. A divergent, putative, specialized transcription factor ApiAP2 has also been reported in the apicomplexa [36]. A large percentage of proteins in *T. gondii *are 'hypothetical proteins' with no known function and might possibly encode parasite-specific functions, including transcriptional regulatory proteins. It is plausible that such highly divergent regulatory proteins utilize very different *cis*-elements for their recruitment, which would explain the absence of canonical *cis*-elements in the promoters studied thus far.

We have exploited the availability of genome sequence for *T. gondii *to identify conserved upstream motifs in diverse groups of functionally related genes. We identified over-represented motifs by *de novo *pattern finding and tested their function *in vitro*, in the parasite, by specifically mutagenizing them in their native promoter context and measuring reporter activity. For each group, two candidate motifs were selected and characterized for their function in their endogenous promoter. We find that seven out of eight motifs identified by *de novo *pattern finding show a statistically significant role in promoter activity. We have shown that conserved over-represented motifs play a definite role in gene-expression, and can affect promoter activity either positively or negatively (Figures 1, 2, 3, 4). It is exciting to note that some of the motifs that affect gene expression also exhibit cross-species conservation, as shown by their presence upstream of orthologs in *E. tenella *(Table 1).

Our studies have shown that in spite of the lack of *a priori *knowledge concerning the nature of regulatory sequences and/or expression profiles, it is possible to identify putative *cis*-regulatory elements. We have shown that elements identified purely on the basis of computational techniques can be functionally relevant for gene expression. We previously characterized putative *cis*-regulatory elements in the genome of *C. parvum *in a similar fashion where we established a correlation between over-represented elements and co-ordinate gene expression [37]. In a comparison of the genes common to both studies (genes of the glycolytic and nucleotide metabolism pathways), we do not detect identical motifs in *T. gondii *and *C. parvum*. Given the evolutionary divergence and difference in genome organization and content between these two parasites, it is not unexpected that they do not share some specialized components of the regulatory machinery, or these components may have evolved so rapidly as to be unrecognizable as orthologs.

The use of asynchronous populations of parasites, as is the case here, is expected to dilute out expression effects to some extent, and this is reflected in the occasional small, but significant, change in promoter activity upon mutagenesis of a motif (Figure 1d, motif GLYCA; *p *< 0.05). In spite of these limitations, our study has successfully identified six novel *cis*-regulatory elements and established the functional significance of one previously reported conserved upstream element. The study of stage-specific gene regulation in *T. gondii *has been an active area of investigation, and small-scale microarray studies have reported genes that are preferentially expressed in either developmental stage [38,39]. Recently, three *T. gondii *studies have reported the presence of novel *cis*-regulatory elements in promoters of genes regulated in a stage-specific manner [10-12]. However, gene regulation within the tachyzoite stage has not been well studied. The lack of a synchronized population of tachyzoites (with respect to their cell cycle) in *in vitro *culture makes it difficult to address gene regulatory questions in the actively multiplying tachyzoite, as any given population of cells in culture consists of parasites at different points of their cell cycle.

Our study reports the presence of different *cis*-regulatory elements controlling gene-expression within the tachyzoite stage of the parasite and is among the first to show evidence for the presence of modular organization of promoters in *T. gondii*. Using site-specific mutagenesis of conserved upstream motifs and subsequent reporter assays, we have shown that the identified *cis*-elements can exhibit co-operative, additive or redundant effects within a promoter and operate in a sequence-specific manner to control gene expression (Figures 1, 2, 3, 4). The putative transcription factors or repressors recruited by these elements in order to facilitate gene regulation remain to be determined. One of the limitations of investigating gene expression within the tachyzoite stage is the lack of a parasite population enhanced in the production of a specific transcription factor that could be recruited by these *cis*-regulatory elements. Consequently, the detection of such putative transcription factors or repressor proteins from a mixed population of parasites by experiments such as electrophoretic mobility shift assays has proven to be challenging and yield inconsistent results (data not shown).

## Conclusions

*Cis*-regulatory elements play a significant role in gene regulation in *T. gondii *and can operate individually and in concert to influence gene expression. This study provides a glimpse of the extent and mechanisms by which *cis*-regulatory elements are involved in controlling gene expression within the tachyzoite life cycle of *T. gondii*. We have shown evidence for upstream elements to behave as both positive and negative regulators of gene expression as well as exhibit redundancy within the same promoter in downstream gene expression. One of the eight motifs examined in this study, the TRP-2 (RPB) motif, is similar in sequence to the binding site for ApiAP2, a family of apicomplexan-specific DNA-binding proteins. *De novo *computational approaches possess great predictive power in compact genomes when sequence is available. We have shown here the applicability of computational techniques to identify gene regulatory signals in a system where little is known about gene regulation.

## Materials and methods

### Computational analyses

Whole genome sequence (v.3.3) and gene-predictions for *T. gondii *were obtained from ToxoDB (release 4.0) [40]. Scripts were written in PERL to extract the upstream sequence (2 kb or until the previous upstream gene is encountered, whichever sequence was smaller) for every predicted gene to create an 'upstream sequence database'. This database was screened for possible missed protein coding regions by performing a BLASTX against all protein sequences in the non-redundant database at NCBI. Sequences that contained greater than 80% similarity to coding sequences were pruned. Groups of genes (hereafter referred to as seed genes) used to identify common over-represented upstream motifs were selected based on the hypothesis that genes belonging to the same biochemical or functional pathway should be co-regulated and, hence, possess common upstream regulatory elements. Members from each of the four groups considered in this study (glycolysis, nucleotide metabolism, micronemal proteins and ribosomal proteins) were identified based on existing annotation from ToxoDB. Additionally, BLAST analyses using sequence information of orthologs from *P. falciparum *and *C. parvum *were employed to identify the corresponding genes in *T. gondii *when necessary. Given the limited annotation at the time of this study, it is possible that the gene lists under each functional group are not exhaustive. The pattern-finding algorithm MEME [15] was used to identify over-represented conserved motifs in the upstream regions of genes within each functional group. MEME was run using the parameters minw = 8, maxw = 20, in all three modes (tcm, oops and zoops) and the results were manually examined to pick candidate motifs. Upstream sequences of corresponding orthologs from *E. tenella *(a related coccidian parasite) were also used whenever possible to identify evolutionarily conserved motifs. In order to narrow down the results of MEME, a rule-based approach was adopted to pick candidate motifs for subsequent experimental validation. Candidate motifs included those that were over-represented in the upstream regions in comparison to the background set (entire upstream regions database), showed considerable conservation in sequence and/or position, and were present in all the sequences within each group.

The program MAST [15] was used to search the most recently annotated version of the *T. gondii *genome (release 4.3) for the presence of each candidate motif in all 7,817 genes in the genome divided into coding regions (16,862,741 bp) and upstream regions (11,685,162 bp). To normalize for the different sizes of the coding regions database and the upstream regions database, the motif density was computed by calculating the number of motifs per 10,000 bp. Chi square analysis was performed to examine whether there was a significant difference in occurrence of each motif between upstream and coding regions.

The expected frequency of motifs within each set (the upstream regions database and the coding regions database) was calculated as follows. The upstream regions database has a base composition of 48% AT (A = 23%, T = 25%, G = 25% and C = 26%) and the coding regions database has a base composition of 42% AT (A = 22%, T = 20%, G = 30%, C = 28%). Given these statistics, the 8 bp motif GLYCA (GCTKCMTY) will be expected to occur once every 1,780 bases in the upstream regions database and once every 2,406 bases in the coding regions database (after accounting for positional degeneracy). Taking the database sizes into account, the expected motif density per 10,000 bases was calculated for each motif in each database. Chi square analysis was used to examine the statistical significance of the differences in the observed and expected frequencies of motifs in the upstream and coding regions.

#### Assessment of enrichment of GO categories within genes containing a candidate motif

Of the 7,817 predicted genes in *T. gondii*, 2,437 genes have been assigned the subcategory cellular component, 3,902 genes have been assigned the subcategory molecular function, and 4,738 genes have been assigned the subcategory biological process. First-level GO annotations from each of these subcategories (totaling 44 subcategories) for all motif-containing genes were obtained from ToxoDB along with the total number of genes in the genome corresponding to each annotation. Hypergeometric probability distribution was used to determine the chance probability of observing the number of genes with a given GO annotation within each of the eight sets of candidate upstream regulatory motif-containing genes compared to the number of genes with that GO annotation in the whole genome. More specifically, the probability of observing at least *x *genes that contain an upstream motif with a given annotation in a random subset of *n *genes is given by [41]:



where *A *is the total number of genes with a particular GO annotation and *N *is the total number of genes within the genome (7,817). In order to control error rates for multiple hypothesis testing, we applied two distinct methods, the Benjamini Hochberg method [42] and the Bonferroni method [43] (Table 4 in Additional data file 1). In the case of the Benjamini Hochberg method, a false discovery rate criteria based on the number of GO terms searched (number of tests = 44 GO terms searched × 8 motifs = 352) was implemented and a false discovery rate adjusted *p*-value < 0.05 was considered significant.

Additionally, specific, lower-level GO categories that describe the functional groups chosen in this study (for example, glycolysis [GO:0006096], ATP-binding [GO:0005524], nucleoside metabolism [GO:00009116], calcium binding: [GO:0005509], translation: [GO:0006412] and ribosome structure: [GO:0003735]) were picked to similarly test for their enrichment within the motif-containing gene sets. Significant enrichment of any of these within the corresponding motif-containing gene sets was determined using hypergeometric probability.

### Molecular techniques

For each group of functionally related genes considered in this study, a promoter that contained a single occurrence of the candidate motif was chosen to study the role of the motif in driving gene expression. Promoter sequences were PCR-amplified from parasite genomic DNA and a two-step overlap-extension PCR technique was employed to carry out site-directed mutagenesis [44] to alter the candidate motif sequence (see Table 1 in Additional data file 1 for primer sequences). All or a majority of the bases in each motif were substituted by base-specific transversions, thus destroying the original sequence of the candidate motif but maintaining the spacing within the promoter (Figures 1c, 2c, 3c and 4c). Successful mutagenesis was confirmed by sequencing or by restriction digest analysis. The Gateway™ cloning system was used to clone the WT and mutagenized promoters individually upstream of a firefly luciferase-expressing vector (test-firefly). As an internal control, a constitutive promoter (*T. gondii *α-tubulin promoter)-driven renilla luciferase-expressing construct (α-tub-renilla) was co-transfected along with the experimental construct (Additional data file 2).

### Parasite culture and transient transfections

*T. gondii *RH tachyzoites were cultured in human foreskin fibroblasts (hTERT cells, BJ Biomedicals) as previously described [8]. Transient transfection was performed via electroporation, using freshly lysed parasites, needle-passaged and filtered through a 3 micron filter and resuspended in cytomix [8,45]. Immediately prior to use, freshly prepared 2 mM ATP and 5 mM glutathione were added to the cytomix and sterile-filtered. For each co-transfection, 2 × 10^7 ^parasites were transfected via electroporation with a mixture of sterile circular plasmid DNA of α-tub-renilla (control) and test-firefly mixed in a ratio of 2.5:1. (40 μg test + 16 μg control). Electroporation was performed in a 2 mm gap cuvette using a BTX electroporator: 1.8 kv, 100 Ω, 25 μF. Post-electroporation, the parasites were allowed to rest for 15 minutes in the cuvette and then transferred to T25 tissue culture flasks. Then, 18-24 hours post-electroporation, the cells were scraped and lysed using passive lysis buffer (Promega, Madison, WI, USA) and a dual luciferase assay was performed with the extract using the Promega Dual Luciferase kit. Briefly, the different substrate requirements for each enzyme, firefly luciferase and renilla luciferase allowed us to assay reporter expression for each construct sequentially within the same extract. Reporter activity from the WT or mutagenized promoter was measured relative to the internal control, eliminating errors due to variation in parasite populations and individual transfections. Enzyme activity was measured using a dual luciferase-ready luminometer. Each electroporation experiment was performed in triplicate and luciferase assays were performed in duplicate for expression measurements. The unpaired Students *t*-test was used to calculate the statistically significant difference in expression levels between WT and mutagenized promoter activity; *p *< 0.05 was considered statistically significant.

## Abbreviations

ENO: enolase; GO: Gene Ontology; LDH: lactate dehydrogenase; MAST: Motif Analysis and Search Tool; MEME: Multiple Em for Motif Elicitation; RPL9: Ribosomal protein L9; UPRT: uracil phospho-ribosyl transferase; WT: wild type.

## Authors' contributions

NM and JCK conceptualized the study, NM conducted the analyses and wrote the manuscript and JCK provided advice and revisions to the manuscript. SJ conducted statistical analyses to address motif over-representation and GO enrichment.

## Additional data files

The following additional data are available with the online version of this paper: an Excel spreadsheet with supplementary Tables 1-4 (Additional data file 1); a PDF figure explaining the dual transfection and luciferase assay experimental set up (Additional data file 2).

## Supplementary Material

Additional File 1 Table 1: oligonucleotide sequences used in Gateway™ cloning and site-directed mutagenesis. Table 2: genome-wide list of genes that contain a candidate regulatory motif in their upstream region. Table 3: comparison of expected and observed number of motifs genome-wide in upstream and coding regions. Table 4: significant GO category enrichments (raw *p*-value < 0.05) within each motif-containing gene set. Click here for file

Additional File 2 The dual transfection and luciferase assay experimental set up. Click here for file
