# Supplementary figures and images for: Identification and functional characterization of cis-regulatory elements in the apicomplexan parasite Toxoplasma gondii
Source: Genome Biol. 2009 Apr 7;10(4):R34. doi: 10.1186/gb-2009-10-4-r34 (PMC2688925; doi:10.1186/gb-2009-10-4-r34)

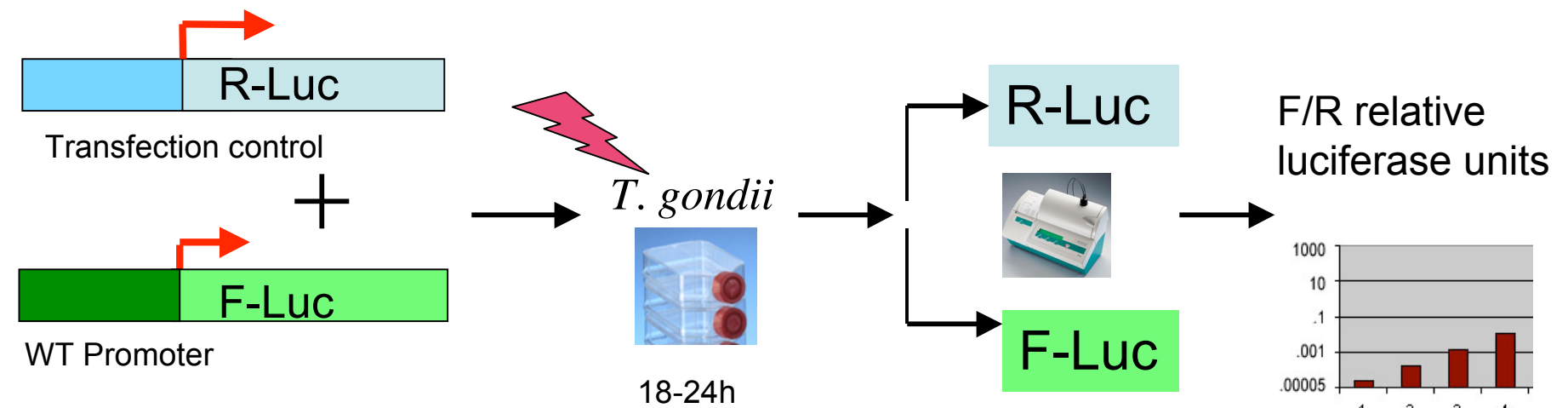

A

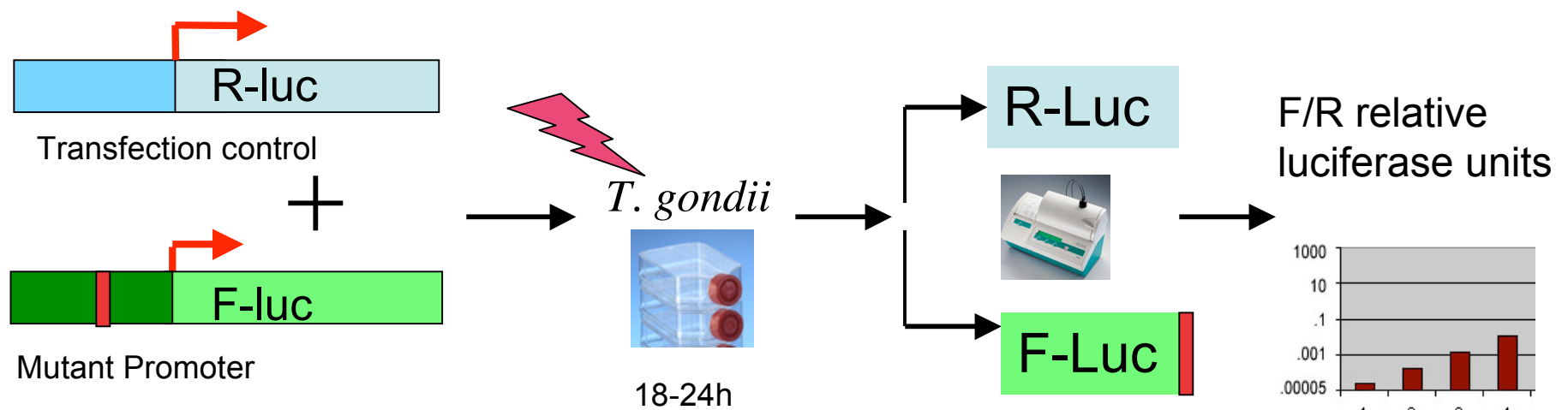

B

Supplement: Additional File 2 — The dual transfection and luciferase assay experimental set up. [file gb-2009-10-4-r34-S2.pdf]
